# Porphyrias, porphyrins and hepatocellular cancer.

**DOI:** 10.1038/bjc.1986.159

**Published:** 1986-07

**Authors:** N. O. Bengtsson, L. Hardell


*Br. J. Cancer* (1986), **54**, 115–117

### Short Communication

### Porphyrias, porphyrins and hepatocellular cancer

N.O. Bengtsson & L. Hardell

*Department of Oncology, University Hospital, S-901 85 Umeå, Sweden*

Disturbances of heme synthesis, and thereby porphyrin metabolism and excretion, are well known in patients with chronic liver disease of various aetiology such as heavy metal exposure, liver cirrhosis and chronic hepatitis (Doss & Martini, 1977). Also in bone marrow diseases such as leukaemia, pernicious and haemolytic anaemias such disturbances have been reported. The usual findings in liver and bone marrow diseases have been that of an asymptomatic patient with isolated coproporphyrinuria. A number of case reports have been published on patients with liver tumours, benign as well as malignant including liver metastases, coincident with symptomatic hepatic porphyria clinically manifest as porphyria cutanea tarda (PCT) (Thompson *et al.*, 1970; Waddington, 1972). In some of these cases a reduced uroporphyrinogen decarboxylase activity has been detected and this enzyme defect has been regarded as a paraneoplastic phenomenon caused by liver damage (Doss & Martini, 1977). An increased risk of hepatocellular cancer (HCC) has been reported in patients with PCT (Berman & Braun, 1962; Kordac, 1972; Salata *et al.*, 1985). Liver cancer in PCT patients has almost invariably developed in cirrhotic livers, cirrhosis thought to be the prerequisite for carcinogenesis. Agents associated with an increased risk of HCC such as alcohol, steroid hormones and aflatoxin are potent porphyrinogenic substances (Kappas & Granick, 1968; Sherlock, 1981; Zawirska & Bednarz, 1981). In animal experimental models of porphyria hexachlorobenzene (HCB) has been widely used (Koss *et al.*, 1983; Wainstok de Calmanovici *et al.*, 1984). HCB is also a liver carcinogen in several animal species (Cabral *et al.*, 1977; 1979; Smith & Cabral, 1980).

Recently an association between porphyria acuta intermittens (PAI) and HCC has been reported (Lithner & Wetterberg, 1984; Hardell *et al.*, 1984; Kaupinnen & Mustajoki, Unpublished). In our case control study 3 cases of PAI were identified among the 83 patients with HCC *versus* none among the 200 controls (Hardell *et al.*, 1984). PAI is an inborn error of metabolism inherited as a dominant trait and characterized by a reduced activity of the rate-limiting enzyme in heme synthesis, uroporphyrinogen-1-synthetase. The disease is often manifested at the age of puberty. The clinical picture of an acute attack is characterized by abdominal pain, neurological symptoms, changes in electrolytes and marked increase in the excretion of two precursors in heme synthesis, amino levulinic acid (ALA) and porphobilinogen (PBG). PAI is a rare disease worldwide and our region is actually one of the high incidence areas with an estimated prevalence of 1 case per 1000 inhabitants (Waldenström, 1969).

A mapping of the pedigrees of 2 families has been performed and is presented in Figures 1a, b.

In total 5 cases of HCC in persons with PAI were identified in these 2 families. The age at diagnosis was: 66, 67, 69, 69 and 70 years. The male in the first generation in family 1 was the carrier of

**Figure 1a, b** Pedigrees of 2 families with porphyria acuta intermittens. PAI = porphyria acuta intermittens; HCC = hepatocellular cancer.

Correspondence: N.O. Bengtsson.
Received 2 January 1986; and in revised form, 17 March 1986.

© The Macmillan Press Ltd., 1986

the trait. He died at the age of 77 from bronchopneumonia, autopsy was not performed. In family 1 there were 4 siblings with PAI in the second generation of whom 1 died at the age of 43 in an acute attack of porphyria and the other three developed HCC. In family 2 the female in the first generation was the carrier of the trait. She died at the age of 46 of uraemia. Of the 5 siblings in the second generation 3 had PAI of which 2 developed HCC. The third person with PAI died at the age of 46 with an extrahepatic cholangiocellular carcinoma. No further cases of HCC have been found in the 2 families but the living carriers of the trait are still fairly young. In all cases of HCC in this pedigree mapping, as well as in the case control study (Hardell *et al.*, 1984), diagnoses were based on histopathological examination of biopsy and autopsy material.

Various hypotheses can be postulated for the accumulation of HCC in families with PAI. The genetic locus for uroporphyrinogen-1-synthetase is located to the long arm of chromosome 11 (Meisler *et al.*, 1980). This point mutation could be associated with an oncogene responsible for the high risk of HCC development in these persons. No data are available regarding this, however. Another theory is that porphyrins are carcinogenic *per se*. This hypothesis can be supported by:

1. Porphyrins have photochemical cytotoxic properties and upon illumination form highly reactive oxygen radicals (Weishaupt *et al.*, 1976; Brault *et al.*, 1985). This effect has been used to treat cutaneous and subcutaneous metastases of various cancers in humans (Wile *et al.*, 1984).
2. In a recent study of HCB-induced hepatocarcinogenesis rats were fed HCB in diet for 90 weeks (Smith *et al.*, 1985). Both sexes showed a decrease in uroporphyrinogen decarboxylase activity. Massive porphyria developed in females but not in males. After 90 weeks of HCB treatment 100% of the female rats developed multiple liver tumours *versus* only 16% of the male rats. Analysis of HCB content in liver tissue showed no differences between males and females. These findings indicate that HCB exerts its hepatocarcinogenic action via porphyrins.
3. In a study of patients with PCT 17 out of 342 patients followed in the years 1954–69 developed HCC. Another group of 367 PCT patients were treated with chloroquine 250 mg p.o. twice a week and followed in the years 1969–83. Of these only 3 developed HCC. The cutaneous symptoms as well as the urine excretion of uroporphyrin and coproporphyrin were significantly lowered in the chloroquine group (Kordac *et al.*, unpublished).
4. In a study of porphyrin metabolism a group of patients with liver cirrhosis and another group with liver cirrhosis and HCC were compared with healthy controls (Udagawa *et al.*, 1984). A significant increase of uroporphyrin and coproporphyrin excretion in urine was found in both patient groups. Furthermore HCC patients showed significantly higher excretion of porphyrins compared with patients with only liver cirrhosis. No correlation between porphyrin excretion and liver function tests was found.

The increased risk for HCC in persons with hepatic porphyrias and results from experimental investigations have initiated further studies on porphyrins in hepatocarcinogenesis.

### References

BERMAN, J. & BRAUN, A. (1962). Incidence of hepatoma in porphyria cutanea tarda. *Rev. Czech. med.*, **8**, 290.

BRAULT, D., NETA, P. & PATTERSSON, L.K. (1985). The lipid peroxidation model for halogenated hydrocarbon toxicity. Kinetics of peroxyl radical processes involving fatty acids and Fe(III)porphyrins. *Chemico-Biol. Res.*, **54**, 289.

CABRAL, J.R.P., SHUBIK, P., MOLLNER, T. & RAITANO, F. (1977). Carcinogenic activity of hexachlorobenzene in hamsters. *Nature*, **269**, 510.

CABRAL, J.R.P., MOLLNER, T., RAITANO, F. & SHUBIK, P. (1979). Carcinogenesis of hexachlorobenzene in mice. *Int. J. Cancer*, **23**, 47.

DOSS, M. & MARTINI, G.A. (1977). Porphyrin metabolism and liver tumors. In *Primary liver tumors*, Renner *et al.* (eds) Falk Symposium 25, Titisee, West Germany, October, 1977.

HARDELL, L., BENGTSSON, N.O., JONSSON, U., ERIKSSON, S. & LARSSON, L.G. (1984). Aetiological aspects on primary liver cancer with special regard to alcohol, organic solvents and acute intermittent porphyria—an epidemiological investigation. *Br. J. Cancer.*, **50**, 389.

KAPPAS, A. & GRANICK, S. (1968). Experimental hepatic porphyria. Studies with steroids of physiological origin in man. *Ann. N.Y. Acad. Sci.*, **151**, 842.

KORDAC, V. (1972). Frequency of occurrence of hepatocellular carcinoma in patients with porphyria cutanea tarda in long-term follow-up. *Neoplasma*, **19**, 135.

KOSS, G., SEUBERT, S., SEIDEL, J., KORANSKY, W. & IPPEN, H. (1983). Studies on the toxicology of hexachlorobenzene. V. Different phases of porphyria during and after treatment. *Arch. Toxicol.*, **52**, 13.

LITHNER, F. & WETTERBERG, L. (1984). Hepatocellular carcinoma in patients with acute intermittent porphyria. *Acta Med. Scand.*, **215**, 272.

MEISLER, M., WANNER, L., EDDY, R.E. & SHOWS, T.B. (1980). The UPS locus encoding uroporphyrinogen 1 synthase is located on human chromosome 11. *Biochem. Biophys. Res. Commun.*, **95**, 170.

SALATA, H., CORTÉS, J.M., ENRÍQUEZ DE SALAMANCA, R., OLIVA, H., CASTRO, A., KUSAK, E., CARRENO, V. & HERNANDEZ GUÍO, C. (1985). Porphyria cutanea tarda and hepatocellular cancer. Frequency of occurrence and related factors. *J. Hepatol.*, **5**, 477.

SHERLOCK, S. (1981). Hepatic porphyria. In *Disease of the liver and biliary system.* Sherlock (ed) p. 381. Blackwell Scientific Publications: Oxford.

SMITH, A.G. & CABRAL, J.R.P. (1980). Liver-cell tumors in rats fed hexachlorobenzene. *Cancer Lett.*, **11**, 169.

SMITH, A.G., FRANCIS, J.E., DINSDALE, D., MANSON, M.M. & CABRAL, J.R.P. (1985). Hepatocarcinogenecity of hexachlorobenzene in rats and the sex difference in hepatic iron status and development of porphyria. *Carcinogenesis*, **6**, 631.

THOMPSON, R.P.H., NICHOLSON, D.C., FARNAN, T., WHITMORE, D.N. & WILLIAMS, B. (1970). Cutaneous porphyria due to a malignant primary hepatoma. *Gastroenterology*, **59**, 779.

UDAGAWA, M., HORIE, Y. & HIRAYAMA, C. (1984). Abberant porphyrin metabolism in hepatocellular carcinoma. *Biochem. Med.*, **31**, 131.

WADDINGTON, R.T. (1972). A case of primary liver tumor associated with porphyria. *Br. J. Surg.*, **59**, 653.

WAINSTOK, DE CALMANOVICI, R., DEL C. RIOS DE MOLINA, M.C., TAIRA DE YAMASATO, M., TOMIO, J.M. & C. SAN MARTIN DE VIALE, L. (1984). Mechanism of hexachlorobenzene-induced porphyria in rats. *Biochem. J.*, **218**, 753.

WALDENSTRÖM, J. (1969). Porphyria. In *Medicinskt Kompendium II.* Iversen *et al.* (eds) p. 1132. Arnold Busck, Nyt Nordisk Forlag: Köpenhavn.

WEISHAUPT, K.R., GOMER, C.J. & DOUGHERTY, T.J. (1976). Identification of singlet oxygen as the cytotoxic agent in photo-inactivation of a murine tumor. *Cancer Res.*, **36**, 2326.

WILE, A.G., COFFEY, J., NAHABEDIAN, M.Y., BAGHDASSARIAN, R. & MASON, G.R. (1984). Laser photoradiation therapy of cancer: An update of the experience at the University of California, Irvine. *Lasers Surg. Med.*, **4**, 5.

ZAWIRSKA, B. & BEDNAR, W. (1981). The particular traits of carcinogenesis induced in Wistar rats by aflatoxin Bl.III. Porphyrins and the activity of gamma-glutamyl-transpeptidase in primary hepatomas and in their tissue of origin. *Neoplasma*, **28**, 35.


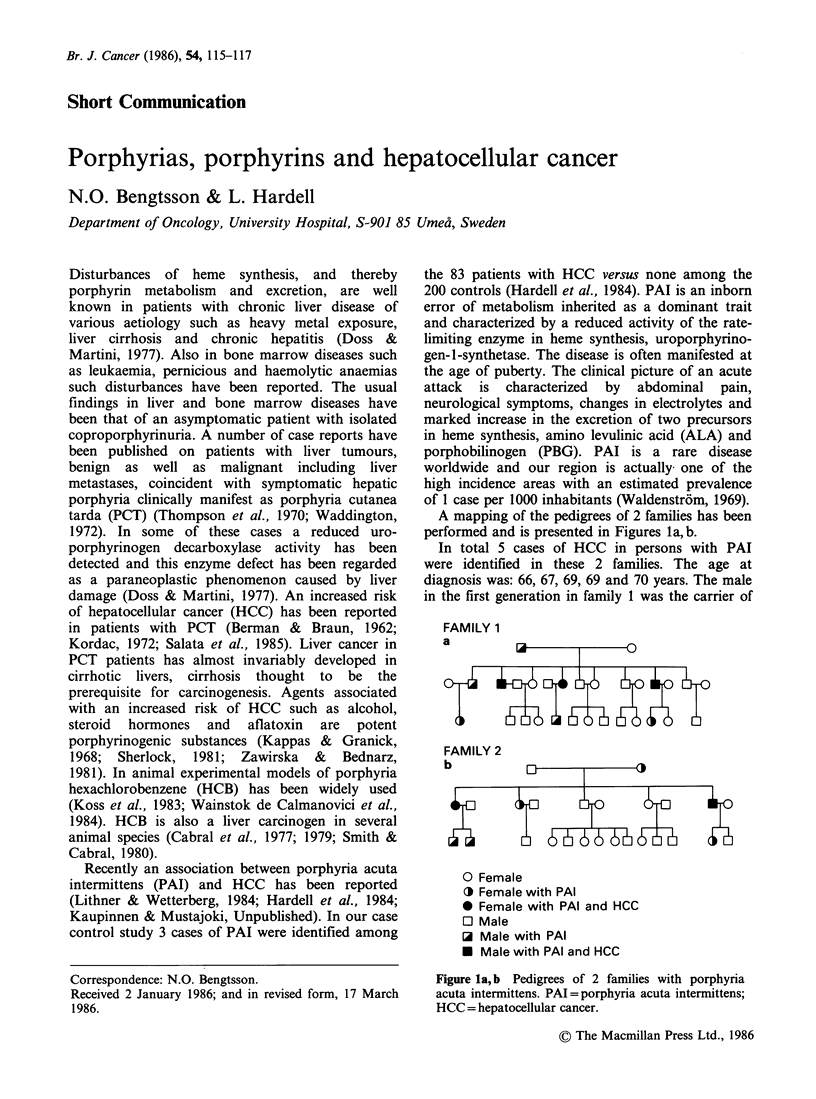

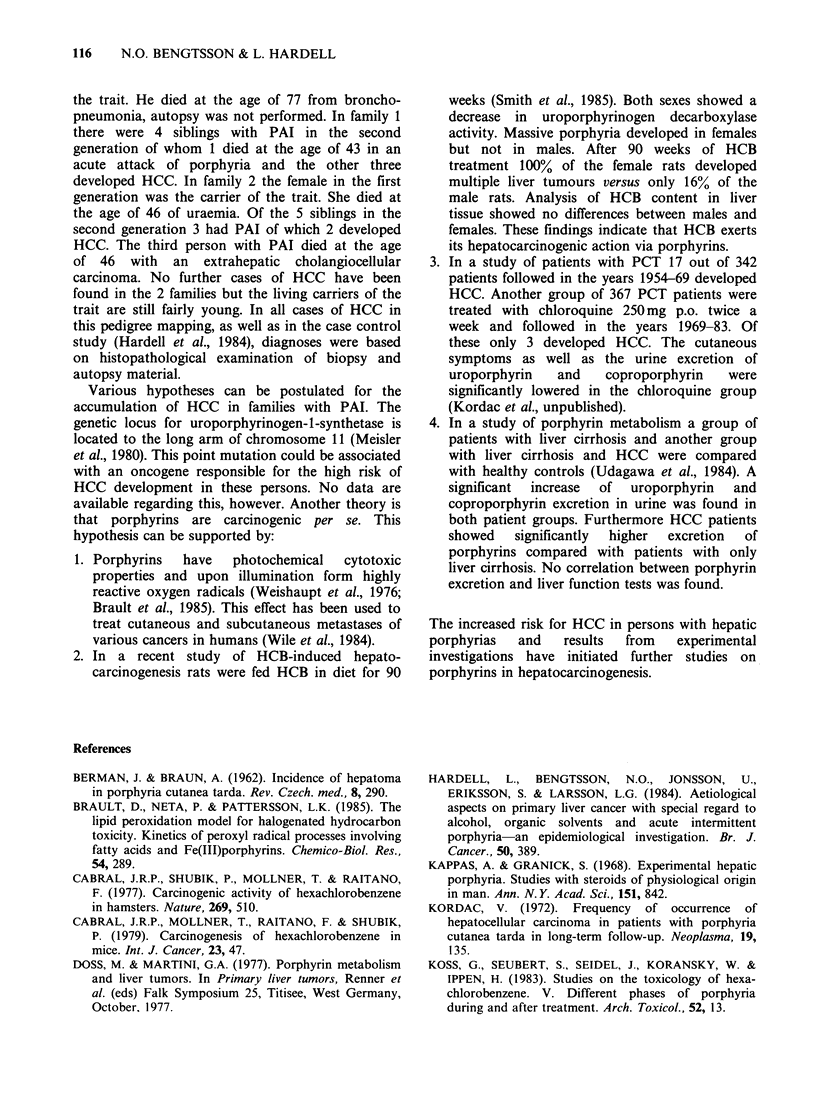

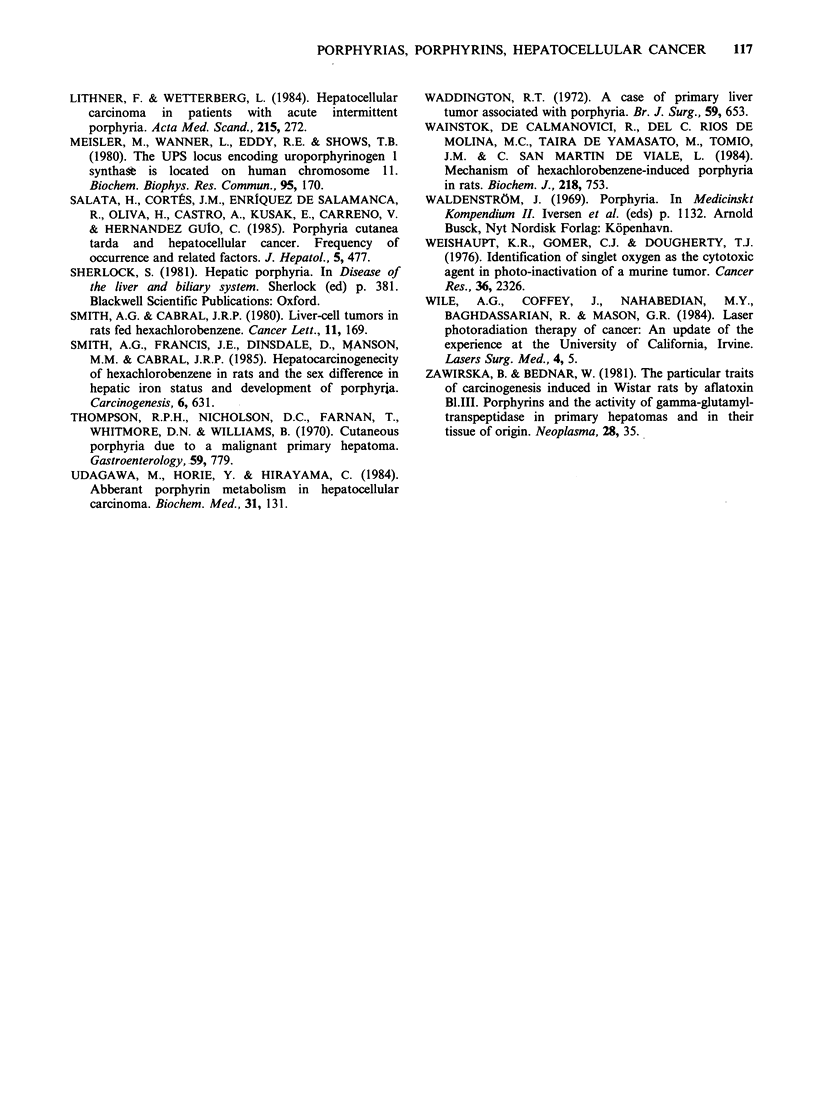


## References

[OCR_00196] BERMAN J., BRAUN A. (1962). Incidence of hepatoma in porphyria cutanea tarda.. Rev Czech Med.

[OCR_00200] Brault D., Neta P., Patterson L. K. (1985). The lipid peroxidation model for halogenated hydrocarbon toxicity. Kinetics of peroxyl radical processes involving fatty acids and Fe(III) porphyrins.. Chem Biol Interact.

[OCR_00212] Cabral J. R., Mollner T., Raitano F., Shubik P. (1979). Carcinogenesis of hexachlorobenzene in mice.. Int J Cancer.

[OCR_00207] Cabral J. R., Shubik P., Mollner T., Raitano F. (1977). Carcinogenic activity of hexacholorobenzene in hamsters.. Nature.

[OCR_00223] Hardell L., Bengtsson N. O., Jonsson U., Eriksson S., Larsson L. G. (1984). Aetiological aspects on primary liver cancer with special regard to alcohol, organic solvents and acute intermittent porphyria--an epidemiological investigation.. Br J Cancer.

[OCR_00231] Kappas A., Granick S. (1968). Experimental hepatic porphyria: studies with steroids of physiological origin in man.. Ann N Y Acad Sci.

[OCR_00236] Kordac V. (1972). Frequency of occurrence of hepatocellular carcinoma in patients with porphyria cutanea tarda in long-term follow-up.. Neoplasma.

[OCR_00242] Koss G., Seubert S., Seubert A., Seidel J., Koransky W., Ippen H. (1983). Studies on the toxicology of hexachlorobenzene. V. Different phases of porphyria during and after treatment.. Arch Toxicol.

[OCR_00255] Meisler M., Wanner L., Eddy R. E., Shows T. B. (1980). The UPS locus encoding uroporphyrinogen I synthase is located on human chromosome 11.. Biochem Biophys Res Commun.

[OCR_00261] Salata H., Cortés J. M., Enríquez de Salamanca R., Oliva H., Castro A., Kusak E., Carreño V., Hernandez Guío C. (1985). Porphyria cutanea tarda and hepatocellular carcinoma. Frequency of occurrence and related factors.. J Hepatol.

[OCR_00273] Smith A. G., Cabral J. R. (1980). Liver-cell tumours in rats fed hexachlorobenzene.. Cancer Lett.

[OCR_00277] Smith A. G., Francis J. E., Dinsdale D., Manson M. M., Cabral J. R. (1985). Hepatocarcinogenicity of hexachlorobenzene in rats and the sex difference in hepatic iron status and development of porphyria.. Carcinogenesis.

[OCR_00284] Thompson R. P., Nicholson D. C., Farnan T., Whitmore D. N., Williams R. (1970). Cutaneous porphyria due to a malignant primary hepatoma.. Gastroenterology.

[OCR_00290] Udagawa M., Horie Y., Hirayama C. (1984). Aberrant porphyrin metabolism in hepatocellular carcinoma.. Biochem Med.

[OCR_00295] Waddington R. T. (1972). A case of primary liver tumour associated with porphyria.. Br J Surg.

[OCR_00299] Wainstok de Calmanovici R., Ríos de Molina M. C., Taira de Yamasato M. C., Tomio J. M., San Martin de Viale L. C. (1984). Mechanism of hexachlorobenzene-induced porphyria in rats. Effect of phenobarbitone pretreatment.. Biochem J.

[OCR_00311] Weishaupt K. R., Gomer C. J., Dougherty T. J. (1976). Identification of singlet oxygen as the cytotoxic agent in photoinactivation of a murine tumor.. Cancer Res.

[OCR_00317] Wile A. G., Coffey J., Nahabedian M. Y., Baghdassarian R., Mason G. R., Berns M. W. (1984). Laser photoradiation therapy of cancer: an update of the experience at the University of California, Irvine.. Lasers Surg Med.

[OCR_00324] Zawirska B., Bednarz W. (1981). The particular traits of carcinogenesis induced in Wistar rats by aflatoxin B1. III. Porphyrins and the activity of gamma-glutamyltranspeptidase in primary hepatomas and in their tissue of origin.. Neoplasma.

